# Emblems and Symbols

**Published:** 1884-10

**Authors:** Ausburn Towner


### THE BISTOURY.

Vol. XX. ELMIRA, N. Y., OCTOBER, 1884. No. 3.

### Contributions.

### EMBLEMS AND SYMBOLS.

AUSBURN TOWNER.

It is somewhat curious to note how large a part in life, not only in these modern days but in the whole history of the world, has been borne by emblems or symbols and in many instances, how much stronger an influence they have than the things themselves of which they are a type or representative. Pictures, paintings or photographs which are but symbols, appeal to the eye with a stronger effect than the scenes or persons that they represent, and the tropes or figures of speech in poetry or prose, fix the attention and enliven the imagination and feelings in a stronger manner than the mere bald statement of a fact or principle. All persons have more or less of the poetic feeling, and even in the commonest conversation, use emblems and symbols, unconciously and parrot-like it may be, but with a full understanding that by such use what they would say is more clearly and the stronger set forth.

There are a great many symbols or emblems in common, every-day life, like the mortar and pestle of the chemist or apothecary, the three golden balls of the pawnbroker, the anvil of the blacksmith and the key of a Treasury that are plain to our comprehension, or whose origin is well-known but there are many others which although we acknowledge their significance we are unable to tell why they should stand for what they do. Take the horseshoe for instance. It is known the world over as a symbol of good luck. Peasants in the ruggedest districts of Ireland place it over the outer doors of their miserable cabins ; and you can see it, gilded, festooned and painted, set in the place of honor on the center table of the most aristocratic mansions on Fifth avenue. The origin of the superstition connecting it with good fortune is lost. Many fanciful stories have been written accounting for it, the multitude of which make it apparent that none of them can be true. The old Rosicrucians had their idea of the matter, and it seems to have been founded on a very logical inference. If theirs be the true solution it hardly speaks well for the dames of fashion to make such a conspicuous display of the object as they do in their parlors and boudoirs. There is the four leaved clover, also, dear to the hearts of the young and innocent of both sexes; and the numerous other sentiments that attach themselves to every specimen of the floral kingdom, the violet, sweet emblem of faithfulness; the white rose, speaking of a withered love; the rose-bud, confession; the daisy, symbol of unconscious beauty; the jasmine, telling of an amiable disposition; the while lily, of purity; the yellow lily of faithfulness and the lily of the valley of delicacy and refinement. These and the multitude of others form emblems that to one knowing of them make the valleys and hillsides a book that may be read, whether with profit or not in this direction, is another question. Whence the origin of such an emblematic language distributed and carried into as much of a scientific detail as any vocabulary made by Cuvier or Humboldt, no one can accurately determine. All through Shakespeare are instances that the language was familiar in his time. Hear sweet Ophelia saying in her madness: "There's rosemary. That's for remembrance,—pray you, love, remember. And there is pansies. That's for thoughts."

We certainly did not get these symbols or emblems from our own rude ancestors of the Teutonic or Saxon stock. Flowers would hardly appeal in such a way to rough men and women who swung battle axes side by side in contests of which Death to one or all was the only termination. Such personalizing of nature goes farther back, even to those somewhat effeminate nations of the Tigris and Euphrates, who gave a soul to every object in nature, and from whom it drifted down through the Greeks and Romans to us. It is pleasant to reflect that perhaps, during the dark ages, such classification was kept alive in the monasteries and convents of Europe whose inmates, besides the offices of their institutions, had little else to occupy their attention. It is to be feared, however, that most of their duties or occupations were hardly as innocent as that which had for its object the care of flowers, or the preservation of their emblematic character.

Other emblems of common or every-day life will readily suggest themselves to every one. Instances of them are seen at every turn, in the newspapers and on the walls of public halls. There is the anchor, that means hope; the heart, standing for faith; the cap of liberty, that at once appeals to our notions of freedom; the circle, indicative of eternity. Most of these best-known symbols have become the property of secret organizations and within their folds, with how much of truth who can say? are taught the meanings of these emblems, and are shown the earliest developments and origins of the signs.

The influence and importance of emblems and symbols becomes more noticable when we pass from the individual to the race or nation. They have borne a conspicuous part in the history of every foremost nation that the world has known. The eagles of Rome or France, the red cross of St. George,—the white plume of Navarre, the "flag of our country," wherever that country is, concentrating as I might say, the force, dignity and power of a nation in one object easily to be perceived and appreciated, are illustrations of the necessity to man's nature for some emblem or symbol that speaks directly to his perceptions although in a roundabout way. A flag, take that of our own country for instance, is often only a piece or pieces of cloth. It is very graceful and attractive to be sure floating in a gentle breeze. In itself it is nothing. It is its emblematic character that gives it all its value.

To appreciate it thoroughly one has need to be deprived of a sight of it for a somewhat lengthy period of time. I had a friend once who by the necessities of his business, was banished from this country to a remote corner in the eastern hemisphere. But little intercourse was had with the United States. Three or four years passed without a word from home, or the sight of a familiar face, and it was not until almost six years after his sojourn in the strange place that one day he was standing on the shore of the harbor and saw a large ship slowly making its way inside. He watched her course for more than an hour, and until she finally swung into position, dropped her anchor and run her flag to the top-mast. It was the star-spangled banner. My friend was beside himself. He danced around on the beach in his ecstacy, shouted and sung in his excitement and finally burst into tears. The emblem of his country spoke to him as forcibly as it does to the soldier at the front, or to the veteran of many battles, although in a different way. One needs, sometimes, such an experience to know fully the force and strength of a mere emblem.

Flags as national symbols are however of comparative recent use, and it may be curious to note what sort of objects have been employed in their places, for that something was necessary is a foregone conclusion. If our friends of the past ages couldn't "rally 'round the flag boys," there must have been some object that spoke to them in just as forcible a manner. We know that each of the tribes of Israel had a special ensign of its own; and that the Egyptians carried into battle images of bulls and crocodiles. This latter should have animated the soldiers to do great deeds of valor if there is anything at all in an emblem. In an engraving in a "Comprehensive Commentary," dear to the memories of my childhood, there appears a number of Assyrians going into battle, their standard being a dove or pigeon. Now, with our notions, no one would think of rallying around a dove for any martial or antagonistic purpose. It is to us an emblem of peace. These Assyrians, however, only showed their loyalty to their queen Semiramis by adopting such a device, her name, surprising as it may seem, meaning, in our language, "dove." Or "dove" being English for Semiramis. Athens also to compliment a woman, or better, a goddess, Minerva, took as its emblem, an owl, that looks wise whether it is or not; Corinth selected a winged horse; Carthage a horse's head, complimentary to Neptune; Persia had the sun; the Germans a serpent and a lion; the Danes three lions and a crow and the Saxons a white horse. All these objects were carved in wood or metal and stood in the place of a flag. We can hardly think that they would appeal to the senses, however, as strongly as does a graceful flag, with a meaning attached to every fold.

There are many other national emblems, of which we in this country know nothing, and it is to be hoped we never will know anything. The crown, for instance. signifying that it is over everything, controlling everything; the sceptre and the globe emblems of power and authority. These symbols coming to mean so decidedly the concentration of power in one person, are not used at all in any of the coats of arms of any of the United States, or by the general government itself, although there are few sovereigns governing by "divine right" who have so much real power as the chief of this nation governing by the will of the people.

These national emblems or symbols might form the subject of an extended and interesting paper, there are so many things they suggest. The character of a nation is shown in them, as our own flag tells plainly what we are, "one from many," numerous distinct forces united in one great people.

There is another view of emblems or symbols that is of peculiar interest, touching as it does, o r religious feelings, and in this direction, the necessity of something of a figurative nature craved by all mankind is very strongly shown, as though the creature could not bear to look square upon the face or form of the Creator, but must see Him through an intermediate helper; as though even sacred things must only be known by an emblem or symbol. This is an idea that permeates through all religions. Priests of idolatrous nations know full well that the idols or stones that they set up for the common herd to worship are only the representatives of the gods they adore, whatever may be the popular notion, and doubtless the first pure worship of the antideluvian world degenerated by ultimately placing the representative, for that which it represented.

Not to go too deep into this abstract and metaphysical view of our subject, but to confine it to the design at first marked out, it is to be observed that one of the strongest and originally most attractive influences of the Christian religion, is the symbolism that has been growing up with it in the past eighteen hundred years. It appeals directly to the feelings and gains a control over the person whether he will or not that tends to lead him in the right direction. Look at the expression: "Lamb of God! Lamb of God that taketh away the sins of the world!" and that other one: "Rock of ages!" The first the symbol of the utmost purity and innocence. The last giving an idea of permanence, even of eternity that is strengthening and full of the most conclusive security. The Christian religion is only just now beginning to feel the force of elements against which in the nature of things, it will find it very difficult to contend. The church militant seems to have little fear of the result, and it is to be hoped such confidence has some foundation, but if it should ever succumb to its adversaries, the very last portion of it to go will be its sublime and beautiful symbolism, as embodied in its hymns and liturgies.

It hardly seems necessary to refer to the emblems and symbols of other religions, they seem so inferior after reflecting upon those belonging to Christianity. Few of them are familiar to the generality of readers and in most instances would have to be explained. There is one conspicuous example, however, that deserves to be named from a popular misapprehension regarding it. This relates to the crescent, indicative of a constant increase, as the moon from which it is copied continues to grow larger until it becomes a perfect globe. This now is considered the peculiar property of the Saracen, the Turk and the Mahometan. On the contrary, it is by no means a purely Ottoman symbol The Turk has stolen that as he stole about all he has got. The crescent was the peculiar mark of the city of Constantinople; it lasted there for centuries, and long before Mahomet himself, was a local and thoroughly Christian emblem. The Turks found it there and adopted it; but they no more invented it than they did the steam engine. In many places in Russia, even now, the crescent is to be seen on churches with the cross above it, the object of their union being to signify the Byzantine origin of the Russian faith. The antithesis of the Crescent and the Cross is therefore altogether an illusion; there is no original hostility between them; the supposed contrast of their meaning has grown up by habit during the last four hundred years but it has no foundation in the genealogy of the crescent.

---

Send $1 to the BISTOURY for Dr. UpdeGraff's book "BODINE'S."

